# Supplementary material for: Open access repository-scale propagated nearest neighbor suspect spectral library for untargeted metabolomics
Source: Nat Commun. 2023 Dec 20;14:8488. doi: 10.1038/s41467-023-44035-y (PMC10733301; doi:10.1038/s41467-023-44035-y)
Supplement: Supplementary file 1 — Supplementary Information [file 41467_2023_44035_MOESM1_ESM.pdf]

Supplementary Information

Open Access Repository-Scale Propagated Nearest Neighbor Suspect Spectral Library for Untargeted Metabolomics

Wout Bittremieux[1,*], Nicole E. Avalon[2], Sydney P. Thomas[3,4], Sarvar A. Kakhkhorov[5,6], Alexander A. Aksenov[3,4,7,8], Paulo Wender P. Gomes[3,4], Christine M. Aceves[9], Andrés Mauricio Caraballo-Rodríguez[3,4], Julia M. Gauglitz[3,4], William H. Gerwick[2,3], Tao Huan[10], Alan K. Jarmusch[3,4,11], Rima F. Kaddurah-Daouk[12,13,14], Kyo Bin Kang[15], Hyun Woo Kim[16], Todor Kondić[17], Helena Mannochio-Russo[3,4,18], Michael J. Meehan[3,4], Alexey V. Melnik[7,8], Louis-Felix Nothias[19,20], Claire O'Donovan[21], Morgan Panitchpakdi[3,4], Daniel Petras[3,4,22,23], Robin Schmid[3,4], Emma L. Schymanski[17], Justin J. J. van der Hooft[4,24], Kelly C. Weldon[3,4], Heejung Yang[25], Shipei Xing[3,4,10], Jasmine Zemlin[3,4], Mingxun Wang[26], Pieter C. Dorrestein[3,4,*]

1. Department of Computer Science, University of Antwerp, 2020 Antwerpen, Belgium
2. Scripps Institution of Oceanography, University of California San Diego, La Jolla, CA 92093, USA
3. Skaggs School of Pharmacy and Pharmaceutical Sciences, University of California San Diego, La Jolla CA 92093, USA
4. Collaborative Mass Spectrometry Innovation Center, University of California San Diego, La Jolla, CA 92093, USA
5. Laboratory of Physical and Chemical Methods of Research, Center for Advanced Technologies, Tashkent 100174, Uzbekistan
6. Department of Food Science, Faculty of Science, University of Copenhagen, Rolighedsvej 26, 1958 Frederiksberg C, Denmark
7. Department of Chemistry, University of Connecticut, Storrs, CT 06269, USA
8. Arome Science inc., Farmington, CT, 06032, USA
9. Department of Immunology and Microbiology, The Scripps Research Institute, La Jolla, CA 92037, USA
10. Department of Chemistry, University of British Columbia, Vancouver, BC V6T 1Z1, Canada
11. Immunity, Inflammation, and Disease Laboratory, Division of Intramural Research, National Institute of Environmental Health Sciences, National Institutes of Health, Research Triangle Park, NC 27709, USA
12. Department of Psychiatry and Behavioral Sciences, Duke University School of Medicine, Durham, NC 27701, USA
13. Department of Medicine, Duke University, Durham, NC 27710, USA
14. Duke Institute of Brain Sciences, Duke University, Durham, NC 27710, USA
15. College of Pharmacy and Research Institute of Pharmaceutical Sciences, Sookmyung Women's University, Seoul 04310, Korea
16. College of Pharmacy and Integrated Research Institute for Drug Development, Dongguk University, Goyang 10326, Korea

17. Luxembourg Centre for Systems Biomedicine, University of Luxembourg, L-4367 Belvaux, Luxembourg
18. Department of Biochemistry and Organic Chemistry, Institute of Chemistry, São Paulo State University, Araraquara, 14800-901, Brazil
19. Université Côte d'Azur, CNRS, ICN, France
20. Interdisciplinary Institute for Artificial Intelligence (3iA) Côte d'Azur, France
21. European Bioinformatics Institute (EMBL-EBI), Wellcome Genome Campus, Hinxton, Cambridge CB10 1SD, United Kingdom
22. Interfaculty Institute of Microbiology and Infection Medicine, University of Tuebingen, 72076 Tuebingen, Germany
23. Department of Biochemistry, University of California Riverside, Riverside, CA 92507, USA
24. Bioinformatics Group, Wageningen University & Research, 6708PB Wageningen, the Netherlands
25. Laboratory of Natural Products Chemistry, College of Pharmacy, Kangwon National University, Chuncheon 24341, Korea
26. Department of Computer Science and Engineering, University of California Riverside, Riverside, CA 92507, USA

Corresponding authors: wout.bittremieux@uantwerpen.be, pdorrestein@health.ucsd.edu

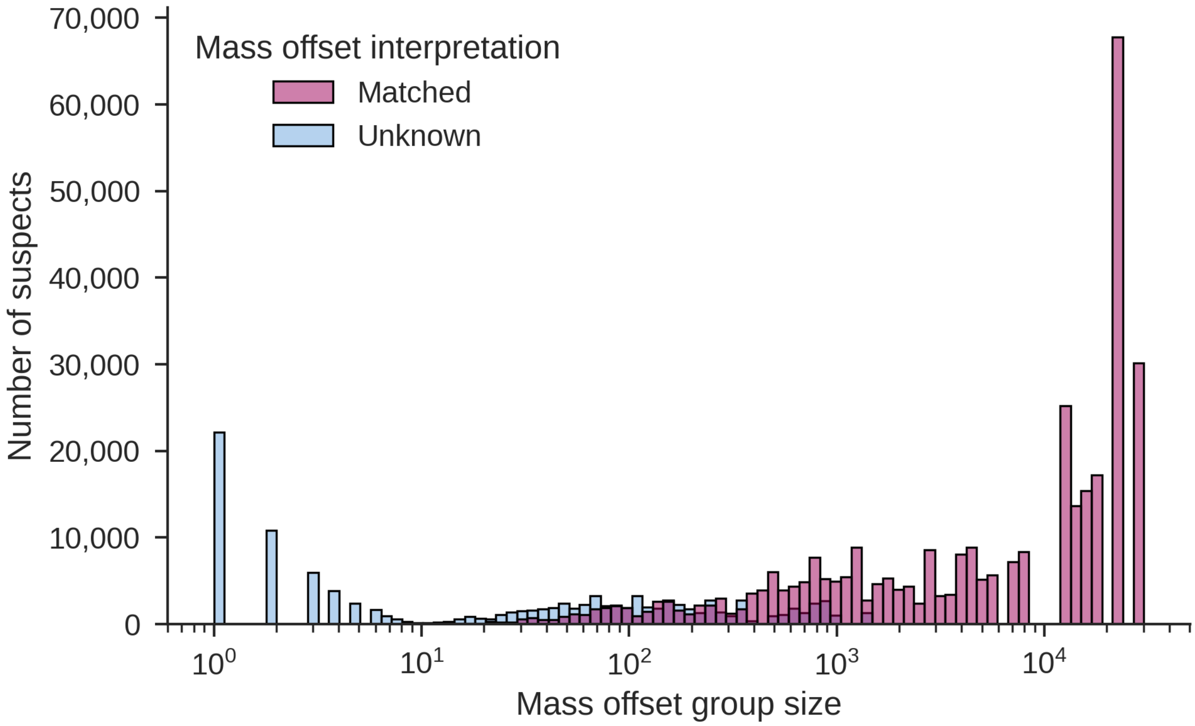

Supplementary Figure 1. Frequency of the observed mass offsets. Several mass offsets occur hundreds to thousands of times, whereas less frequent mass offsets occur only a handful of times. Spectra with delta masses that occur fewer than ten times were not included in the final suspect library. These mass offsets could not be interpreted by matching against modifications in the UNIMOD database[1] and a community curated list of delta masses, and are considered to be non-reproducible mass differences that likely do not correspond to real modifications.

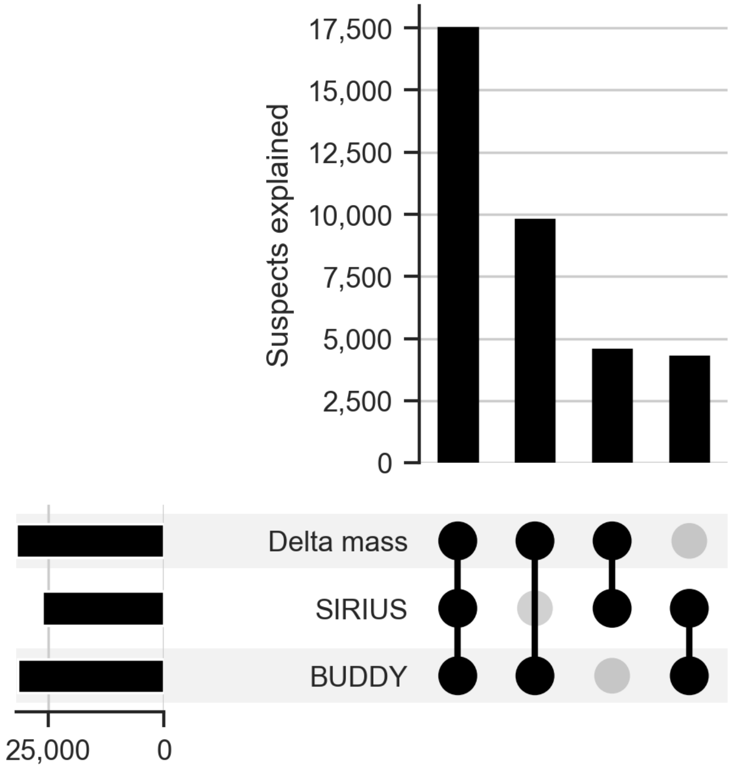

Supplementary Figure 2. Agreement between the delta mass explanations, molecular formulas predicted by SIRIUS,[2] and molecular formulas predicted by BUDDY[3] for suspects for which the molecular formula of the initial molecule is known, that have a valid delta mass explanation, and for which a molecular formula could be predicted by at least SIRIUS or BUDDY. There is a large agreement between the delta mass explanations, SIRIUS, and BUDDY, with only a handful of delta mass explanations that conflict with both the SIRIUS and BUDDY predicted molecular formulas. This indicates that the delta mass explanations and the predicted molecular formulas provide complementary information that can be used to interpret the nearest neighbor suspects.

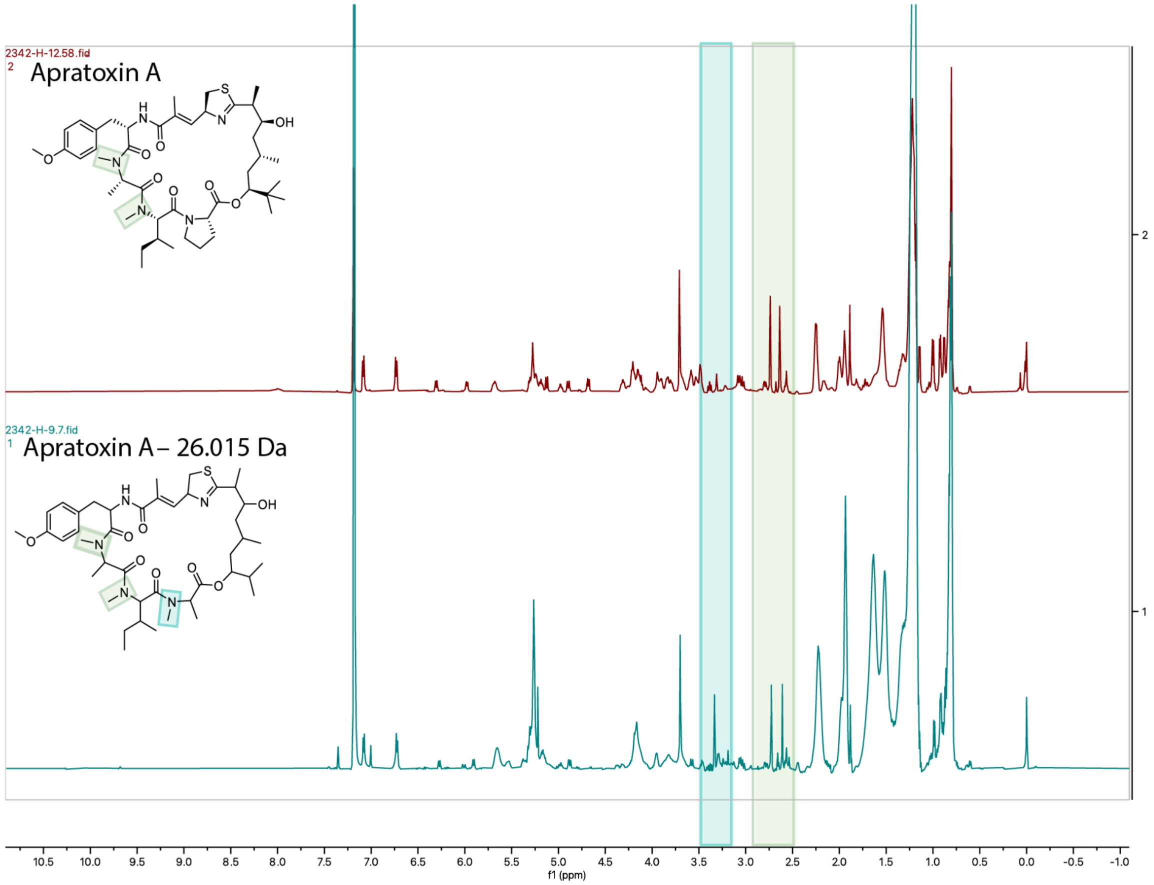

Supplementary Figure 3. Comparison of ^{1}H NMR spectra (600 MHz, $CDCl_3$) of apratoxin A (top) and its related suspect (apratoxin A - 26.015 Da; bottom). Indicated by green shading are the proton signals for the *N*-methyl groups on the *N*-methyl-isoleucine and adjacent *N*-methyl-alanine at 2.71 ppm and 2.81 ppm, respectively. In the suspect there is an additional singlet proton signal observed at 3.41 ppm corresponding to the *N*-methyl-alanine adjacent to the ester bond (turquoise shading). Although the NMR results are consistent with the proposed suspect structure based on the MS/MS data, a full structure assignment was not possible due to the limited and semi-pure sample available for the NMR analysis.

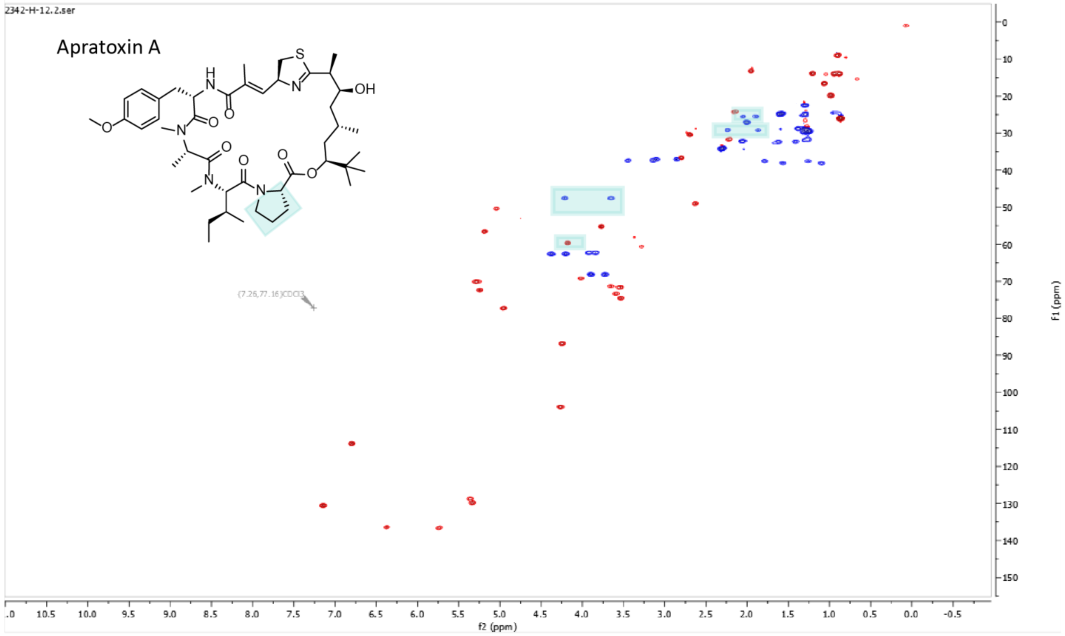

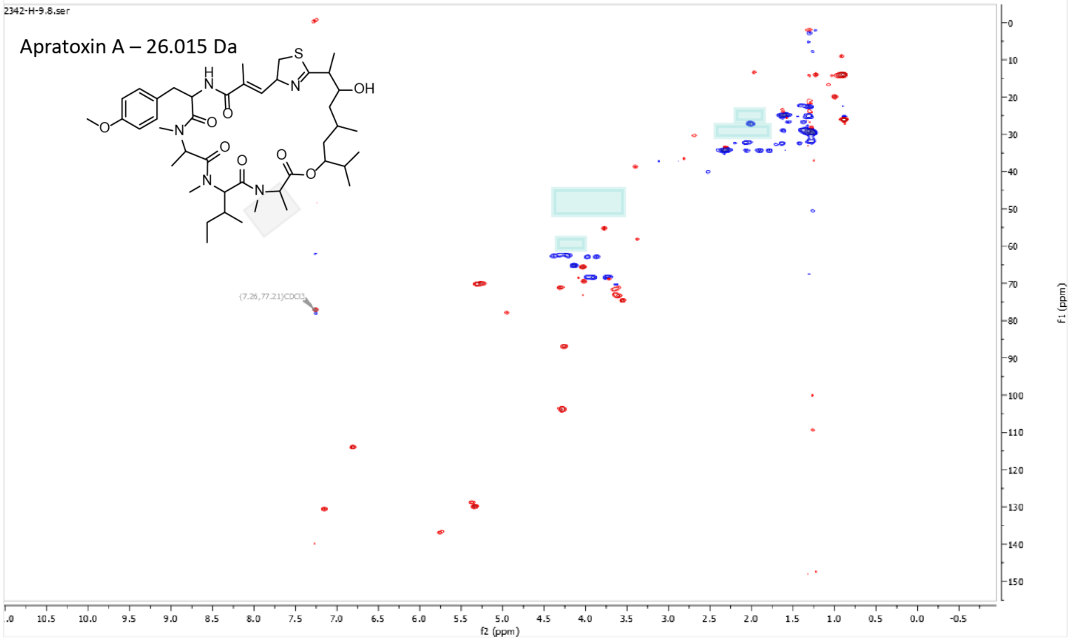

Supplementary Figure 4. Comparison of ^{1}H-^{13}C HSQC spectra (600 MHz, $CDCl_3$) of apratoxin A (top) and its related suspect (apratoxin A - 26.015 Da; bottom). The ^{1}H-^{13}C correlations associated with the proline ring (turquoise boxes) are notably absent in the suspect. Based on the MS/MS fragmentation pattern, the suspect also possesses one less methyl group in the polyketide portion of the molecule: this is possibly explained by an isopropyl rather than a *tert*-butyl group at the initiating terminus, as seen in apratoxin C. Although the NMR results are consistent with the proposed suspect structure based on the MS/MS data, a full structure assignment was not possible due to the limited and semi-pure sample available for the NMR analysis.

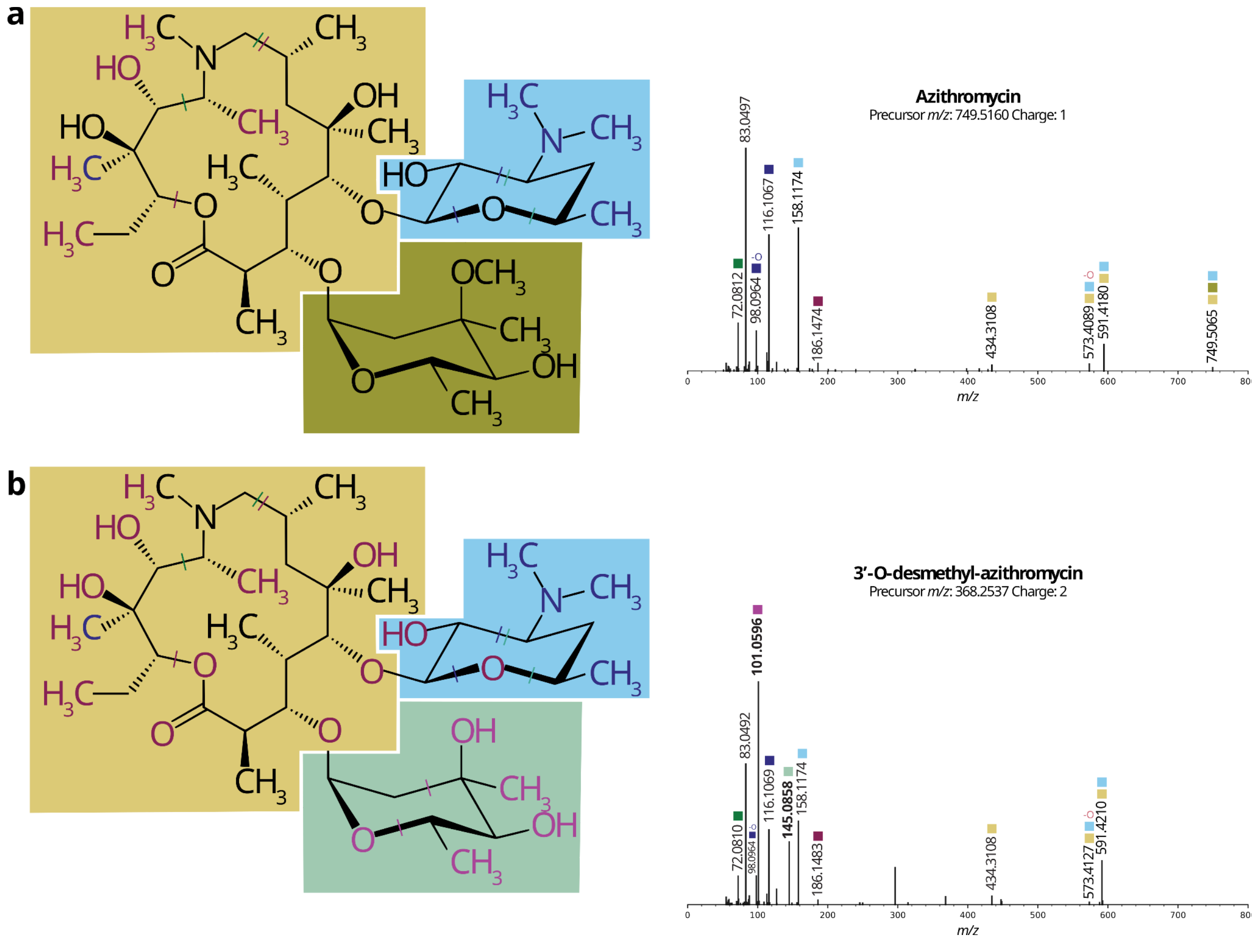

Supplementary Figure 5. Although human breast milk is the gold standard of infant nutrition, the presence of exogenous metabolites—such as food and drugs consumed by the mother—therein is not well understood. This is especially pressing in the case of antibiotics in breast milk, as it is known that antibiotic administration in infancy can cause lasting changes in microbial colonization and host health.[4] A public human breast milk dataset was searched for suspects related to the antibiotic azithromycin (**a**) and found specific azithromycin metabolites, including 3'-*O*-desmethyl-azithromycin (**b**), an azithromycin metabolite previously identified only in snakes.[5]

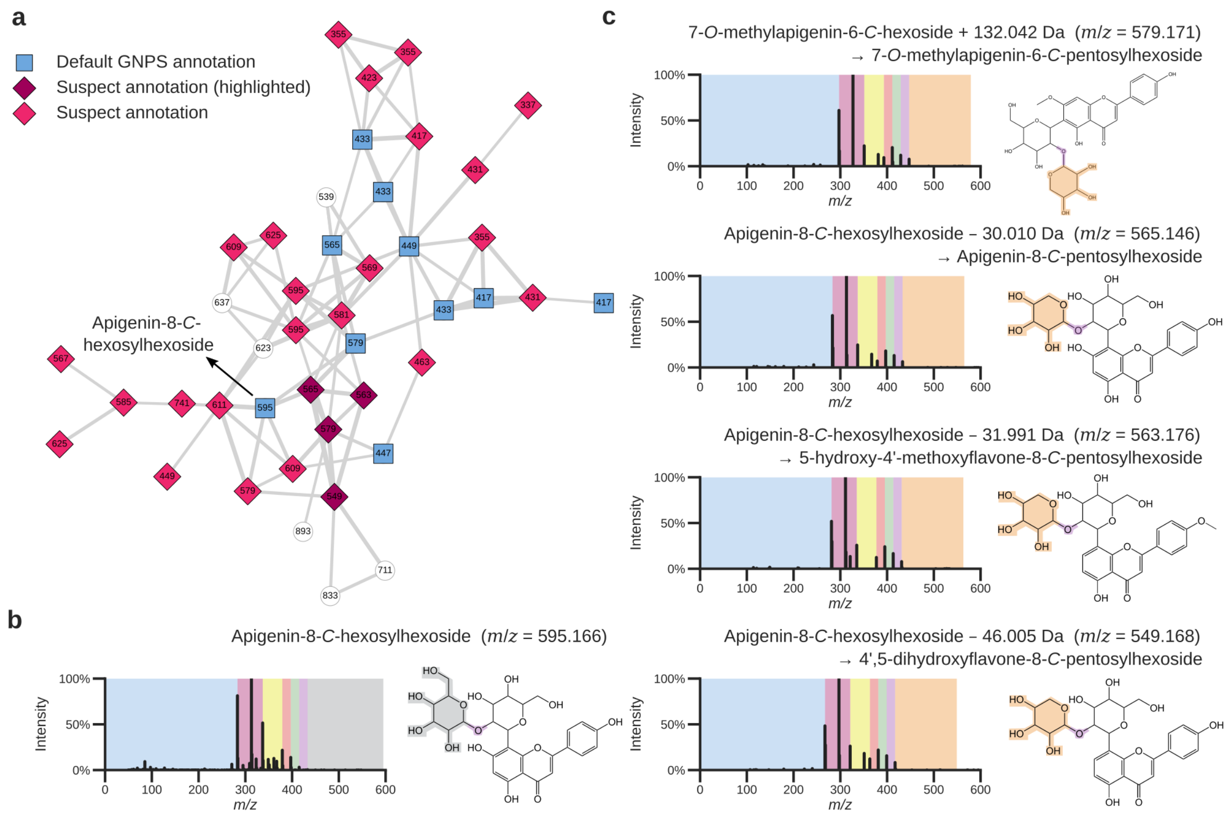

Supplementary Figure 6. Investigation of suspects from a dataset of medicinal plants listed in the Korean Pharmacopeia.[6] **a.** Flavonoids cluster in a molecular network created from the Korean Pharmacopeia medicinal plants dataset. The reference library hits are shown by the blue squares. The purple and pink diamonds are nodes that represent matches to the nearest neighbor suspect spectral library, with the purple diamonds matching the MS/MS spectra shown in panel c for which structures could be proposed. The white nodes are additional MS/MS spectra within the flavonoids molecular family that could not be annotated, even when utilizing the suspect library. **b.** Reference library annotation of an MS/MS spectrum matching to apigenin-8-*C*-hexosylhexoside. **c.** MS/MS spectra and structural hypotheses of apigenin-8-*C*-hexosylhexoside suspects.

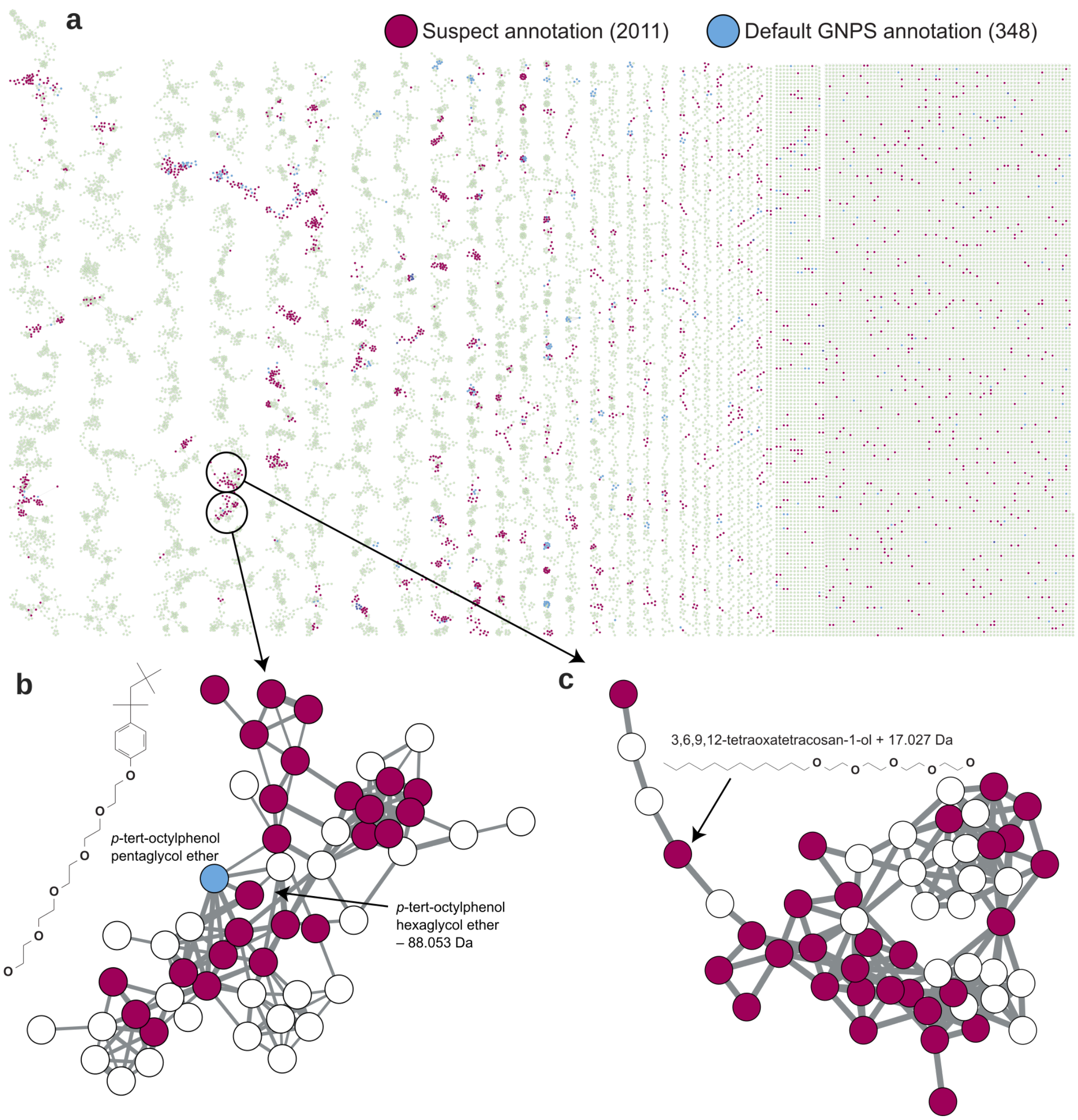

Supplementary Figure 7. Molecular networking of the HOMEChem study to explore the chemistry of a house and how it relates to human activities within.[7] **a.** Inclusion of the suspect library revealed a large portion of the otherwise hidden chemistry, including multiple newly annotated clusters that were found to originate from various skincare-related chemistries, in particular polyether variants. As MS/MS libraries are far from comprehensive, they contain spectra for only a small subset of possible variants of these molecules. This is especially problematic for molecules such as polyethers, as the likelihood of encountering any one particular isomer of many possible variants of polyethers, and related molecules, is very low. **b.** Example of a cluster in the molecular network where multiple spectra could be interpreted based on suspect annotations, while only a single spectrum could be annotated with conventional libraries. **c.** In the majority of cases no annotations were possible at all for skincare ingredient molecules. In contrast, using the suspect library these molecules could be readily identified. All annotations in the cluster are concordant with each other, reinforcing the suspect annotations.

Supplementary References

1. Creasy, D. M. & Cottrell, J. S. Unimod: Protein modifications for mass spectrometry. *PROTEOMICS* **4**, 1534–1536 (2004).

2. Dührkop, K. *et al.* SIRIUS 4: A rapid tool for turning tandem mass spectra into metabolite structure information. *Nat. Methods* **16**, 299–302 (2019).

3. Xing, S., Shen, S., Xu, B., Li, X. & Huan, T. BUDDY: molecular formula discovery via bottom-up MS/MS interrogation. *Nat. Methods* **20**, 881–890 (2023).

4. Aversa, Z. *et al.* Association of infant antibiotic exposure with childhood health outcomes. *Mayo Clin. Proc.* **96**, 66–77 (2020).

5. Hunter, R. P., Koch, D. E., Coke, R. L., Goatley, M. A. & Isaza, R. Azithromycin metabolite identification in plasma, bile, and tissues of the ball python (*Python regius*). *J. Vet. Pharmacol. Ther.* **26**, 117–121 (2003).

6. Kang, K. B. *et al.* Mass spectrometry data on specialized metabolome of medicinal plants used in East Asian traditional medicine. *Sci. Data* **9**, 528 (2022).

7. Aksenov, A. A. *et al.* The molecular impact of life in an indoor environment. *Sci. Adv.* **8**, eabn8016 (2022).
