# Supplementary material for: Open access repository-scale propagated nearest neighbor suspect spectral library for untargeted metabolomics
Source: Nat Commun. 2023 Dec 20;14:8488. doi: 10.1038/s41467-023-44035-y (PMC10733301; doi:10.1038/s41467-023-44035-y)
Supplement: Supplementary file 3 — Description of Additional Supplementary Files [file 41467_2023_44035_MOESM3_ESM.pdf]

Description of Additional Supplementary Files:

Supplementary Data 1: Dataset and file frequency of unique observed suspects

Supplementary Data 2: Frequency of unique observed delta masses and their putative explanations

Supplementary Data 3: Curated list of delta mass interpretations
